# The application of the acoustic shadowing facilitates guidance in radial artery puncture and cannulation teaching in standardized training for residents: a randomized controlled trial

**DOI:** 10.1186/s12909-022-03345-3

**Published:** 2022-04-11

**Authors:** Rui Dong, Jingyan Chen, Hong Wang, Zhilin Liu, Xiaopeng Sun, Yuwei Guo, Mingshan Wang, Lixin Sun, Xiaoping Gu

**Affiliations:** 1 Department of Anesthesiology, Drum Tower Hospital Affiliated to Nanjing University Medical School, No.321 Zhongshan Road, Nanjing, 210008 China; 2 Department of Anesthesiology, Qingdao Municipal Hospital, Qingdao University, Qingdao, 266071 China; 3 Department of Anesthesiology, Qingdao Municipal Hospital, Nanjing Medical University, Qingdao, 266071 China; 4 Department of Education and Training, Qingdao Women and Children’ S Hospital, Qingdao University, Qingdao, 266034 China

**Keywords:** Ultrasound, Acoustic Shadowing, Radial artery puncture, Anesthesiology, Resident standardization training

## Abstract

**Background:**

Radial artery cannulation is a crucial investigative procedure for measuring patients’ blood pressure invasively and serial blood gases. However, radial artery cannulation can be challenging for medical residents, and it is necessary to establish a facile and straightforward teaching strategy. This study aimed to evaluate the efficiency of acoustic shadowing-facilitated ultrasound guidance on radial artery cannulation teaching for medical residents.

**Methods:**

A total of 116 medical postgraduates who underwent standardized residency training programs in the Department of Anesthesiology were randomly divided into a new ultrasound-guided teaching group and a traditional ultrasound-guided teaching group. In the new ultrasound-guided teaching group, radial artery puncture technique was taught by acoustic shadowing-facilitated ultrasound guidance. The training included both theoretical and practical components. After the training, the success rate of the first puncture attempt, the success rate of the catheterization, the ultrasonic positioning time, and the catheterization time of the two groups were compared in a unified manner. A questionnaire on the subjective evaluation of the various aspects of the program by participants was conducted at the end of the training period.

**Results:**

The study included 101 medical residents. The success rate for radial artery puncture at the first attempt in the new ultrasound-guided teaching group was 78.43%. It was significantly higher than that of the traditional ultrasound-guided group (58.00%, odds ratio = 0.380; 95% CI = 0.159 to 0.908; *p* = 0.027). The success rate for the first arterial catheterization in the new ultrasound-guided teaching group was significantly higher than that of the traditional ultrasound-guided group (74.51% *vs.* 52.00%, odds ratio = 0.371; 95% CI = 0.160 to 0.858; *p* = 0.019). The ultrasonic positioning time and catheterization time in minutes in the new ultrasound-guided teaching group were significantly shorter than that of the traditional ultrasound-guided group (14.36 ± 3.31 *vs*. 18.02 ± 4.95, *p* < 0.001; 10.43 ± 2.38 *vs*. 14.78 ± 8.02, *p* = 0.012). However, no significant differences were observed in the incidence of local hematomas and teaching satisfaction scores between the two groups.

**Conclusion:**

Acoustic shadowing facilitates ultrasound-guided radial artery puncture and catheterization is beneficial in the standardized training and teaching of residents. It improves the success rate of the first attempt at radial artery puncture and catheterization and shortens the time of ultrasound location and catheterization.

**Trial registration:**

Registered in the Chinese Clinical Trial Registry on 28 May 2021. Registration number: ChiCTR2100046833.

## Background

Residency training programs are a compulsory part of medical education and a pivotal time in the career path of physicians. In many countries, completion of residency training is a requirement for physicians to practice medicine independently. Residency training is designed to prepare residents for careers as practicing physicians who are capable of meeting patient needs and delivering high-quality clinical care to patients. The quality of resident training is therefore a major issue for healthcare decision-makers and medical educators in medical colleges and teaching hospitals. Postgraduate medical education takes place in most countries in the form of residency training. China has been progressively promoting the standardized residency training programs (SRTP) throughout the country over the past 10 years and in 2014 introduced a new policy to integrate the training for clinical postgraduates with SRTP [1]. Clinical skills training is an essential part of the SRTP. Research on education methodology for important clinical skills can provide practice and teaching references for standardized training of clinical postgraduates.

Radial artery cannulation is commonly performed in the operating room, intensive care unit, and emergency facilities for invasive arterial pressure monitoring and arterial blood gas analysis to guide clinical diagnosis and treatment. Furthermore, in a standardized training program for residents in anesthesiology departments, this technique requires proficiency by clinicians. Radial artery cannulation is not easy for beginners because of the low caliber of the artery. Multiple failed attempts of cannulation may lead to an arterial hematoma. Ultrasound guidance has emerged as a valuable adjunct to the placement of the radial artery catheter [2]. Undoubtedly, for beginners who are inexperienced in ultrasound and puncture, it is difficult to use ultrasound to guide the puncture of the radial artery [3]. The latest research has shown that acoustic shadowing via double developing lines has more advantages than that of traditional ultrasound guidance in radial artery puncture in children [4]. Not surprisingly, this innovative approach has received a lot of attention from the medical education community [5]. However, whether this method can be quickly mastered by novice residents and improve the success rate of radial artery puncture and catheterization remains to be elucidated. Therefore, this study aimed to compare the success and complication rates of novice residents in radial artery puncture and cannulation using the traditional method and the acoustic shadowing-facilitated ultrasound-guided technology.

## Methods

### Aim

The present study aimed to compare the traditional ultrasound-guided teaching with the acoustic shadowing-facilitated ultrasound-guided teaching and to determine which of the two methods can be more helpful for beginners in learning the radial artery puncture technique.

### Study design

The present study was designed as a single-center, prospective, randomized controlled trial. This study adhered to the applicable CONSORT guidelines and was approved by the Ethics Committee of the Qingdao Municipal Hospital, Qingdao University.

### Study population

All postgraduate students who underwent SRTP in the Anesthesia Department of Qingdao Municipal Hospital were eligible for the study. The inclusion criteria were postgraduate students who had not performed radial artery puncture or received relevant training previously. The sample size was calculated based on our preliminary experiment, in which the success rates of the first puncture in the new ultrasound and traditional groups were 73.91% and 45.45%, respectively. The “two proportions comparison formula” was used to calculate the sample size, with the type I error (α) set to 0.05, the power (1-β) was 0.8, and the sampling ratio was 1. The sample size per group was calculated as 43. Considering a 20% drop-out rate, each group finally contained 52 participants. Given that all the medical postgraduate students signed a training agreement before entering the hospital and that skills training is an inherent requirement of SRTP, no further written informed consent was required as approved by the Ethics Committee.

Patients undergoing an elective surgical procedure that required the use of invasive arterial pressure monitoring, as determined by the attending anesthesiologist, were eligible for inclusion in this study. Exclusion criteria were the following: (1) Age less than 18 years or more than 65 years; (2) BMI ≥ 30 kg/m^2^; (3) negative Allen’s test; (4) patients who had previously undergone multiple arterial punctures or had radial artery cannulation in the previous 30 days; (5) patients with peripheral vascular disease, hemorrhagic shock, atherosclerosis, coagulation disorders, or Raynaud’s disease. Written informed consent was obtained for all patients or their legally authorized representatives.

### Randomization

All students are tested on theoretical medical knowledge after entering the anesthesiology rotation to assess their knowledge base. The involved students were randomly grouped based on entrance examination scores by the stratified randomization method, including the acoustic shadowing-facilitated ultrasound-guided teaching group (ASFU-G) and traditional ultrasound-guided teaching group (TU-G).

### Intervention

The teaching secretary provided the first training on aseptic operation techniques, basic anesthesia operations, and fundamental theories. Specific researchers who have qualifications for standardized training of residents gave theoretical training on radial artery puncture techniques for all students, explaining the anatomical position of the radial artery, puncture preparations, puncture process, and complications. Subsequently, two groups of participants observed the demonstration and explanation in 30 cases of arterial puncture catheterization. Among them, the ASFU-G group learned the radial artery puncture technique under acoustic shadowing-facilitated ultrasound guidance; the TU-G group learned the radial artery puncture technique under traditional ultrasound guidance. In a subsequent period, the two groups of participants performed 30 radial artery puncture placements under the guidance of their respective investigators.

#### Teaching methods in the ASFU-G Group

**Fig. 1:**
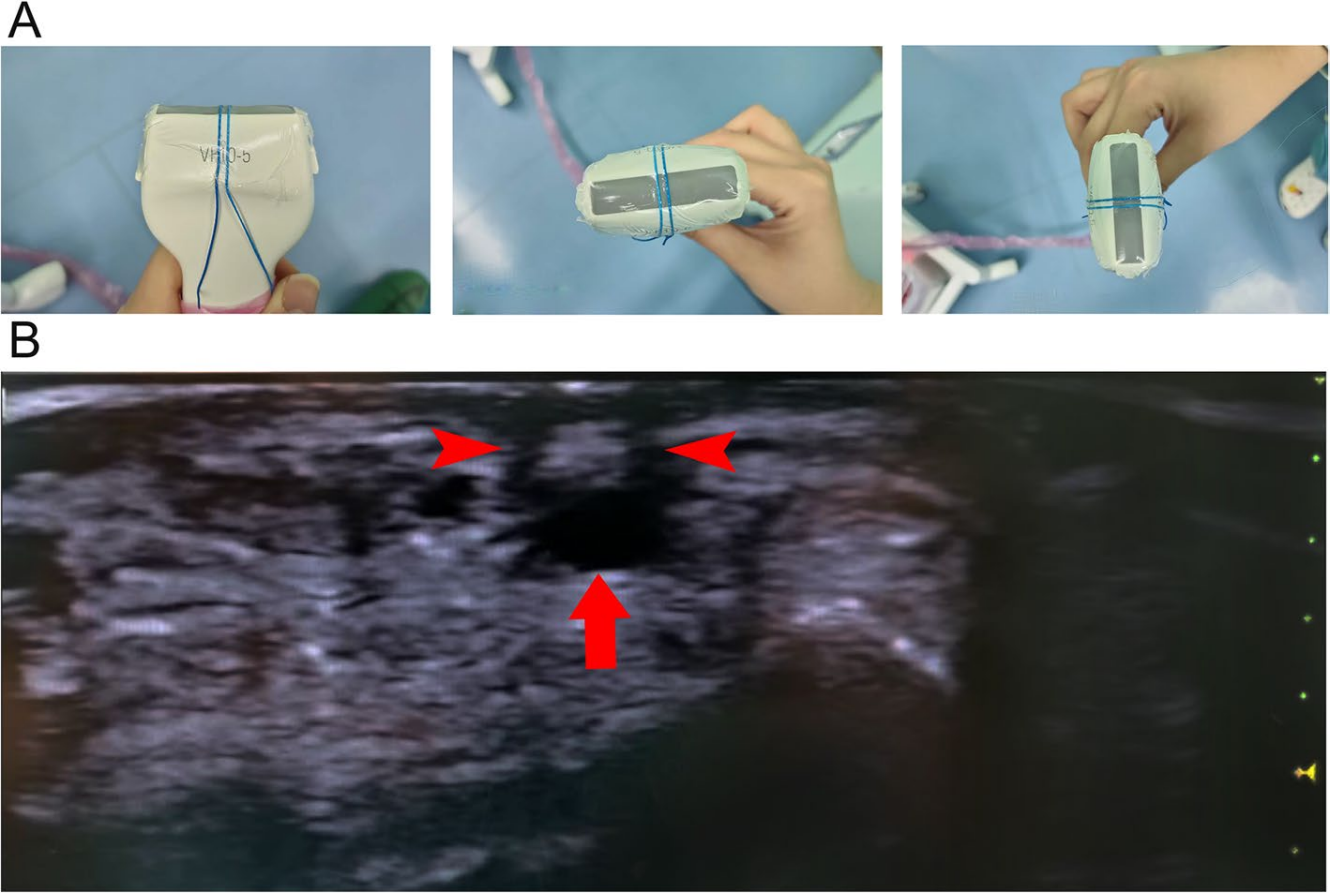
The double developing lines were tied on the ultrasound probe perpendicular to the long axis. **A** Design of ultrasound probe for double developing lines. **B** Ultrasonic image of the radial artery (arrow) and the acoustic shadowing of the double developing line (arrowheads)

**Fig. 2:**
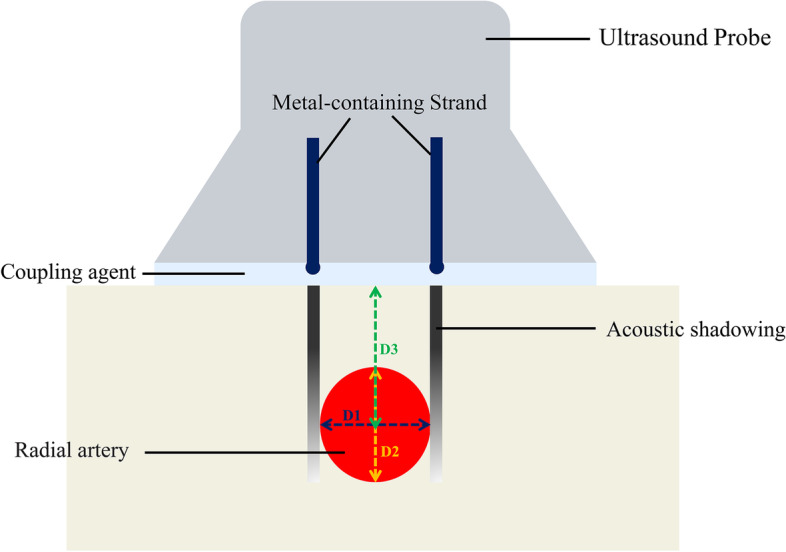
Schematic diagram of the acoustic shadowing of the double developing line. D1 (purple line) represents the transverse diameter of the radial artery; D2 (yellow line) represents the longitudinal diameter of radial artery; D3 (green line) represents the depth from skin to radial artery center

The ultrasound probe modification was conducted by referring to the literature with minor alterations [4]. The metal-containing strand of the X-ray detectable surgical gauze is separated and placed on the ultrasonic probe to make it perpendicular to the long axis of the ultrasonic probe. The ultrasonic coupler and sterile 3 M membrane were applied over the ultrasonic probe to secure the ultrasonic probe and the metal-containing strand, as shown in Fig. 1. The patients were placed in a supine position, with the operative upper limb abducted by 30° with the palm facing upwards and the wrist elevated 3 cm. The skin around the site of the puncture was sterilized with povidone-iodine solution. The ultrasound probe was fitted with sterile disposable plastic caps. The probe, with a frequency of 11 MHz and a depth of 1.5 cm, was adjusted to obtain the highest quality ultrasound. Using the short-axis out-of-plane procedure, the probe was adjusted by moving left and right so that the radial artery was positioned between the middle of the two low-density shadows on the ultrasound image. The central point of the junction between the two visible lines and the skin was the projection point of the radial artery. The needle was inserted at a 30°angle to the skin between the two lines visible on the probe. The tip of the needle, which is located between the low echo acoustic shadows of the visual line, appears as a hyperechoic shadow on the ultrasound screen. Once the needle had entered the radial artery, the angle of the needle was reduced from 30° to 15°, and the needle was slowly pushed forward for 1 to 2 mm. Then the cannula was placed, fixed, and connected to the arterial transducer. The relationship between the developing line and the radial artery on the ultrasound screen is shown in Fig. 2. With the guidance of researchers who explained radial artery anatomy, arterial puncture methods and essentials, causes and analysis of puncture failures, students performed 30 cases of radial artery puncture and catheterization under the guidance of acoustic shadowing-facilitated ultrasound (not limited to the same day).

##### Teaching methods in the TU-G Group

The radial artery puncture and catheterization procedure was performed as described above, but the ultrasound probe was not modified. The radial artery was located by using the short-axis out-of-plane approach. The radial artery puncture and catheterization were performed with the help of color Doppler flow imaging or centerline function (M-line). With the guidance of researchers who explained radial artery anatomy, arterial puncture methods and essentials, causes, and analysis of puncture failures, students performed 30 cases of radial artery puncture and catheterization (not limited to the same day).

### Outcome measures

At the end of the student training, the patients were randomly selected for examination. The patients’ general conditions were recorded. The diameter of the radial artery under ultrasound and the distance from the skin to the center of the internal diameter of the radial artery were measured. The primary endpoint was to compare the success rate of puncture and catheterization upon the first attempt, and the secondary endpoints were ultrasound localization time, puncture time, and the incidence of vascular complications. We defined the ultrasound localization time as beginning with the placement of the ultrasound probe over the skin to the patient’s skin contact with the puncture needle, and the catheterization time was the duration between the contact of the puncture needle with the patient’s skin and the successful insertion of the cannula into the radial artery. All the measured times were documented by a third person. All the measured times were recorded by two researchers and the average values were calculated. A successful first-attempt radial puncture was defined as the entry of the puncture needle into the vessel (ultrasound imaging revealed a puncture needle entering an artery or arterial blood regurgitation within the puncture needle), regardless of the subsequent successful placement of the cannula. However, a successful first-attempt catheterization was defined as not only the puncture needle entering the artery but also the successful placement of the arterial cannula. In the final test, each student had only one opportunity. If the puncture failed, the researcher took over and carried out the subsequent puncture operation. After finishing the experiment, the participants were asked to complete a questionnaire about their satisfaction with the teaching.

### Statistical analysis

Statistical analysis were performed using IBM SPSS Statistics for Windows, version 21.0 (IBM Corp., Armonk, NY, USA). The normality of data was assessed by the Kolmogorov–Smirnov test. The enumeration data are expressed in percentages, the measurement data with normal distribution are expressed as mean ± SD, and the measurement data nonnormally distributed are expressed as median (quartile spacing) values. For two groups with continuous variables, the two-sample independent t-test was used when each set of data was normally distributed with homogeneous variance; otherwise, the Mann–Whitney U test was used. Enumeration data were analyzed with the Chi-square test or Fisher exact probability method. Differences were declared statistically significant if *p* < 0.05.

## Results

**Fig. 3:**
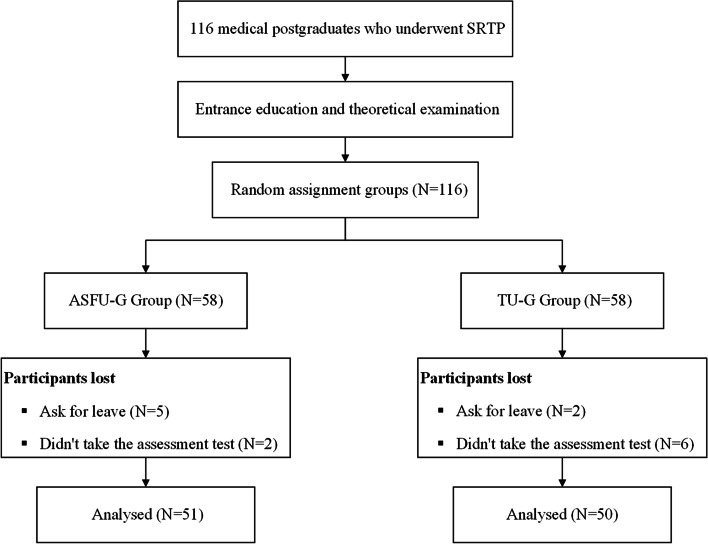
Flow chart for the research

During December 2019 and June 2021, a total of 116 medical postgraduates who underwent SRTP were included in this study. Seven students were unable to complete the training because of leave and eight students failed to participate in the final assessment test, which left 101 students in our final cohort. There were 51 students in the ASFU-G group and 50 students in the TU-G group (Fig. 3). There were no significant differences in age, gender, and entrance examination scores between the two groups (Table 1). In the final skill test, there were no apparent differences in any of the baseline characteristics of the patients that we included between the two groups (Table 2).

**Table 1:** General information of all the medical postgraduates enrolled in the study

Parameter	ASFU-G (*N* = 51)	TU-G (*N* = 50)	*P* value
Age(year)	23.35 ± 0.93	23.10 ± 1.196	0.232
Gender			0.626
Male	19	21	
Female	32	29	
Entrance examination scores	81.96 ± 2.99	82.72 ± 3.58	0.359

Values are mean ± SD or number

**Table 2:** The baseline characteristics of all patients enrolled in the final skills tests

Parameter	ASFU-G (*N* = 51)	TU-G (*N* = 50)	*P* value
Age(year)	65.18 ± 9.63	64.28 ± 11.38	0.670
Weight(kg)	66.43 ± 13.73	68.77 ± 9.47	0.319
Height(cm)	168.69 ± 7.92	167.95 ± 8.61	0.636
ASA grade (I/II/III)	0/37/14	0/39/11	0.526
Gender			0.482
Male	25	28	
Female	26	22	
Heart rate (beats/min)	69.61 ± 7.61	67.20 ± 8.35	0.133
Noninvasive systolic blood pressure (mmHg)	129.53 ± 20.08	130.26 ± 17.55	0.632
Noninvasive diastolic blood pressure (mmHg)	74.22 ± 13.49	77.24 ± 12.41	0.362
Depth from skin to radial artery center (mm)	3.96 ± 1.25	4.37 ± 0.92	0.065
Transverse diameter (mm)	2.49 ± 0.56	2.65 ± 0.59	0.155
Longitudinal diameter (mm)	2.20 ± 0.47	2.17 ± 0.41	0.788

Values are mean ± SD or number

Heart rate, noninvasive systolic and diastolic blood pressures were measured before radial arterial puncture and cannulation

All radial artery diameters are measured at 1.5 cm-2.5 cm on the transverse stripes of the wrist. The measurement is performed during the systolic period in three different cardiac cycles, and the average value is finally taken

As shown in Table 3, compared with the TU-G group, students in the ASFU-G group had a higher success rate in first-attempt radial arterial puncture and catheterization and shorter positioning and catheterization times. The incidence of hematoma was not different between the two groups of patients.

**Table 3:** Comparison of success rate of cannula insertion at first attempt, failure rate, localization time, and puncture time between the two groups

Parameter	ASFU-G	TU-G	Odds Ratio(95%CI)	*P* value
First-attempt puncture success rate	40 (78.43%)	29 (58.00%)	0.380 (0.159–0.908)	0.027
First-attempt catheterization success rate	38 (74.51%)	26 (52.00%)	0.371 (0.160–0.858)	0.019
Ultrasound localization time (s)	14.36 ± 3.31	18.02 ± 4.95	Not applicable	< 0.001
Catheterization time time(s)	10.43 ± 2.38	14.78 ± 8.02	Not applicable	0.012
The incidence of local hematoma	8 (15.69%)	12 (24.00%)	0.589 (0.218–1.594)	0.295

CI Confidence interval

Values are mean ± SD or number (proportion)

A total of 101 questionnaires were distributed in two groups and 101 were recovered, representing a 100% return rate. Before data entry, all questionnaires were checked for logic and integrity. As shown in Table 4, there was no significant difference in the items of the questionnaire and the teaching satisfaction scores between the two groups.

**Table 4:** Summary of the results of the questionnaires

**Entries**	**ASFU-G**			**TU-G**			***P value***
	**YES**	**NO**	**Uncertain**	**YES**	**NO**	**Uncertain**	
Have you improved your clinical skills?	51	0	0	50	0	0	Not applicable
Have you improved your improved learning interests?	51	0	0	47	0	3	0.076
Have you improved theoretical knowledge of ultrasound?	47	0	4	43	2	5	0.320
Have you improved theoretical knowledge of anesthesia?	48	0	3	47	1	2	0.331
Have you mastered radial artery puncture and catheterization?	47	0	4	44	1	5	0.549
Will you recommend this method to others?	49	1	1	46	1	3	0.581
Is it helpful for your clinical work in the future?	47	0	4	45	2	3	0.337
Teaching satisfaction score	9 (8–10)			9 (8–9)			0.475

Values are number or median (quartile spacing)

## Discussions

Arterial line cannulation in patients is traditionally performed by palpation or with Doppler auditory assistance in locating the artery before catheterization. Although the ultrasound technique is more precise than the traditional method, ultrasound technology is more complex for novice practitioners and may require a considerable learning curve for image acquisition, anatomical interpretation, and hand–eye coordination skills. In this study, the application of acoustic shadowing-facilitated ultrasound-guided radial artery puncture was applied to teaching residents about radial artery puncture and catheterization. The primary finding was that acoustic shadowing-facilitated ultrasound-guided radial artery catheterization increases the first-attempt success rate in medical residents. We also found that using acoustic shadowing-facilitated ultrasound-guided radial artery puncture and catheterization can decrease ultrasound location and catheterization time and reduce complications.

Radial artery puncture and catheterization is a common and often difficult task for beginners and even for experienced practitioners. In recent years, visualization technology has provided advantages in improving the success rate of puncture and catheterization. Previous studies have found that the application of ultrasound-guided radial artery puncture can effectively enhance the learning efficiency and training quality of novice resident physicians [6, 7]. Some studies have found that even for physicians without experience in puncture, ultrasound-guided radial artery puncture and catheterization can significantly improve the success rate of radial artery puncture at the first attempt and shorten the puncture time [8–10], particularly in children [4]. Success at the first attempt is crucial during radial artery cannulation, as vasospasm or hematoma resulting from failed attempts further decreases the internal diameter of the artery. Our study suggested that acoustic shadowing-facilitated ultrasound-guided approach can obviously improve the success rate of puncture and catheterization of the radial artery on the first attempt. While this pilot study was limited to an anesthesiology training program, a significant opportunity exists to expand educational precepting experiences across all spheres of residency training, given the similarities in precepting models across many disciplines.

The double-developing lines technique helps locate the projection point of the radial artery on the skin surface to enable quick and accurate determination of the puncture point, and the direction of the puncture needle can be kept at the same level with the help of a bilateral acoustic shadow puncture. This method is different from the traditional ultrasound method that judges the needle route subjectively. It can determine the puncture point and the puncture direction with the help of the double-developing lines technique and has a strong objective standard. After determining the puncture point, the direction of the needle is maintained at the same level as that of the developing lines of the ultrasound probe, and it is easy to obtain the image of the vertical direction of the needle, which helps shorten the puncture time. Another advantage of this method is that the operator can focus on the two low-density shadows instead of staring at the entire screen of the ultrasound monitor. Although this novel modified procedure also requires an ultrasound, it does not require a skilled ultrasound operator to identify the site of puncture [11]. Moreover, compared with the unmodified smooth ultrasound probe, the developing lines attached to the probe can fix the puncture needle, which is also the reason for the high success rate of radial artery puncture and catheterization in the ASFU-G group. Therefore, this technology is theoretically suitable for personnel with no extensive academic theoretical and contextual knowledge of ultrasound.

Female and hypotension are factors in the failure of radial artery puncture and catheterization [12, 13], which may be related to the smaller radial artery diameter. For example, in Japan, it has been reported that the diameter of the distal radial artery was 2.04 ± 0.43 mm in men and 1.96 ± 0.44 mm in women [14]. The depth of the radial artery from the skin also influences the success rate of radial artery puncture and catheterization. A study in pediatric patients revealed that when the depth of the radial artery is 2–4 mm from the skin, the success rate of the radial artery puncture technique under ultrasound guidance is high [15]. In fact, being too close to the skin is not conducive to improving the probability of successful ultrasound-guided radial artery puncture. This is probably because the ultrasound shows the needle tip only when it reaches a certain depth [15]. In our study, there was no significant difference in gender, blood pressure, and the diameter and depth of radial artery among the two groups of patients. Thus, the difficulty level in the final skill test was equal for the two groups.

There are a variety of existing advanced ultrasound imaging devices that can be used as auxiliary to radial artery puncture technology, such as electromagnetic navigation ultrasound [16]. However, the distribution of medical resources in China is relatively imbalanced, and most medical resources are concentrated in medical centers and provincial medical institutions [17–19]. The reality of the situation is that not all the students receive SRTP in large teaching hospitals with abundant medical resources. Therefore, it may be difficult to implement these procedures that rely on advanced devices in other teaching hospitals in low-resource settings. In addition to standard ultrasound equipment, the acoustic shadowing-facilitated ultrasound guidance technology adopted in this study that does not need other high-value equipment, and it is easy to operate and suitable for the teaching residents, further promoting homogenization of SRTP training.

This study has some limitations. First, to ensure the homogeneity of educational interventions, these data were obtained at a single urban teaching hospital, and validation from other institutions is needed. Second, the ultrasound probe modification is time-consuming. The modification of the ultrasonic probe was prepared by the instructor in advance. Undoubtedly, it will take longer if the probe is modified by students themselves. Third, patient assignment was not entirely random, because only patients who were willing to participate were included. Although we primarily included homogeneous surgical patients suitable for participation in this study, our process cannot guarantee that all students will be taught and tested on the exact same radial artery. Finally, the use of Doppler-assisted and M-line-assisted approaches in the TU-G group may have improved the success rate of radial artery puncture and catheterization with the traditional short-axis approach to some extent, and we should be aware of the possibility of this bias.

## Conclusions

Using acoustic shadowing-facilitated ultrasound-guided radial artery puncture and catheterization in the standardized teaching of residents can help students master the skills of radial artery puncture and catheterization more quickly and increase the first-attempt success rate. We therefore recommend this teaching method.

## Data Availability

The datasets generated and analyzed during the current study are not publicly available due to limitations of ethical approval involving the patient data and the confidentiality of student information but are available from the corresponding author on reasonable request.

## Abbreviations

SRTP: Standardized residency training programs
ASA: American Society of Anesthesiologists
BMI: Body mass index
